# Supplementary material for: Comparison of Enhanced Photocatalytic Degradation Efficiency and Toxicity Evaluations of CeO2 Nanoparticles Synthesized Through Double-Modulation
Source: Nanomaterials (Basel). 2020 Aug 6;10(8):1543. doi: 10.3390/nano10081543 (PMC7466517; doi:10.3390/nano10081543)
Supplement: Supplementary file 1 [file nanomaterials-10-01543-s001.pdf]

nanomaterials

Supplementary Information

Comparison of Enhanced Photocatalytic Degradation Efficiency and Toxicity Evaluations of CeO_2 Nanoparticles Synthesized Through Double-Modulation

Jang Hyun Choi [1], Jung-A Hong [2], Ye Rim Son [3], Jian Wang [4], Hyun Sung Kim [3,*], Hansol Lee [1,*] and Hangil Lee [2,*]

1 Department of Biological Sciences, College of Natural Science, Inha University, 100 Inha-ro, Michuhol-gu, Incheon 22212, Korea; jhchoi3@inha.edu
2 Department of Chemistry, Sookmyung Women's University, Seoul 04310, Korea; junga4540@naver.com
3 Department of Chemistry, Pukyong National University, Busan 48513, Korea; syl75218@daum.net
4 Canadian Light Source and University of Saskatchewan, 44 Innovation Boulevard, Saskatoon, Saskatchewan S7N 2 V3, Canada; Jian.Wang@lightsource.ca
* Correspondence: kimhs75@pknu.ac.kr (H.S.K.); hlee@inha.ac.kr (H.L.); easyscan@sookmyung.ac.kr (H.L.); Tel.: +82-2-710-9409 (Hangil Lee)

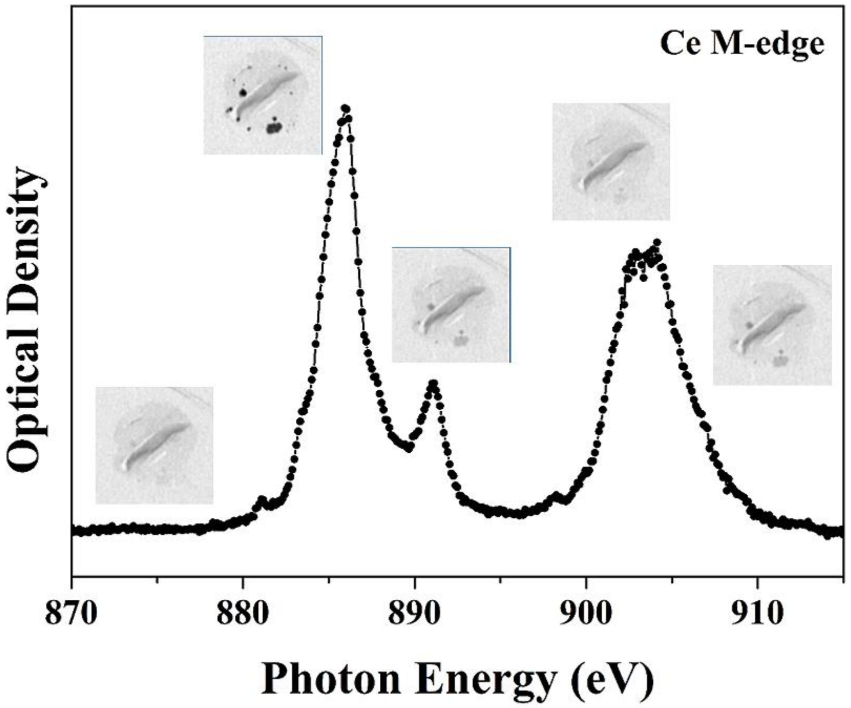

Figure S1. Confirmation of NPs. XAS spectrum and the STXM images depending on the photon energy.

Figure S1 shows a change in the intensity of Cr@CeO_x NPs according to the fixed photon energy. As shown in the figure, it can be seen that the intensity of nanoparticles in each stack image also changes according to the intensity of the Ce M-edge peak.

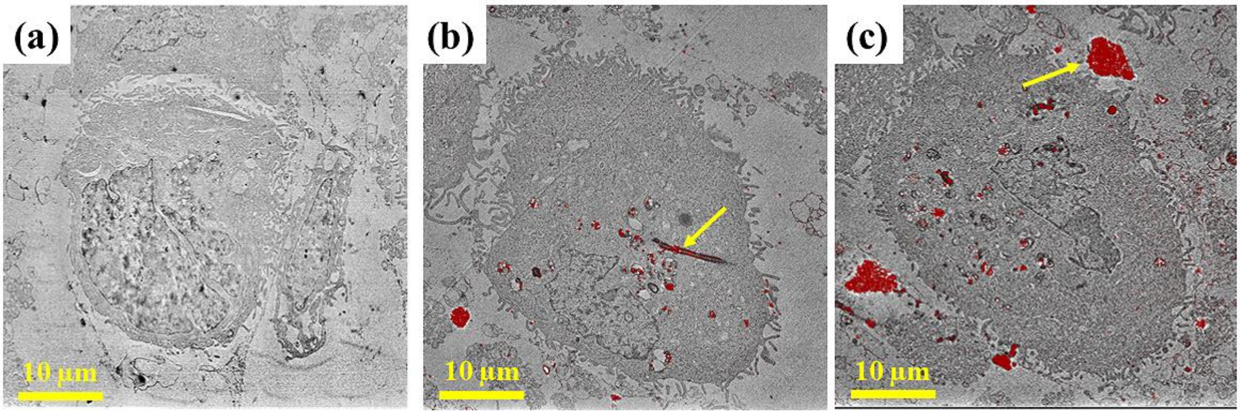

Figure S2. NPs tracking methods. (**a**) Control (no NPs image), (**b**) Cr@CeO$_x$, and (**c**) Fe@CeO$_x$ NPs penetrated bio-TEM images. * Red color NPs is modified CeO_2 NPs. Arrow indicates the position of nanoparticles.
